# Children’s Environmental Health Indicators for Pacific Island Countries

**DOI:** 10.3390/ijerph15071403

**Published:** 2018-07-03

**Authors:** Claire Brereton, Amelia Turagabeci, Donald Wilson, Peter D. Sly, Paul Jagals

**Affiliations:** 1 Children’s Health and Environment Programme, Centre for Children’s Health Research, University of Queensland, Brisbane, QLD 4101, Australia; claire.brereton@uq.edu.au (C.B.); p.sly@uq.edu.au (P.D.S.); 2 School of Public Health and Primary Care, Fiji National University, Suva, Fiji; amelia.turagabeci@fnu.ac.fj (A.T.); donald.wilson@fnu.ac.fj (D.W.)

**Keywords:** children’s environmental health indicators (CEHI), CEH, CEHI, EHI, Pacific Island Countries (PICs), Healthy Islands

## Abstract

Healthy environments support the wellbeing of children and the environment thus play a cardinal role in the future of Pacific Island Countries (PICs). Children are more vulnerable and at risk to environmental hazards than adults because they breathe, drink, and eat much more relative to body weight, resulting in greater exposures in the different environments in which children find themselves every day. We examine the role that children’s environmental health indicators (CEHI) can play for PICs to highlight priorities and we prioritise actions to improve children’s environmental health and thus achieve their ‘Healthy Islands’ vision. We conducted a systematic search of relevant documented and publicly available Pacific Island Country information on children’s environmental health indicators using the general Internet, as well as databases such as PubMed, Google Scholar, relevant UN agencies, as well as regional databases. Information on CEHI was available—mainly in grey literature—but not specifically aimed at PICs. Likewise, similar observations were made for peer-reviewed literature. From this review, we compiled summaries and a framework to propose the requirements as well as provide a foundation for the development of CEHI for PICs. CEHI development for PICs should ideally be a multi-sectoral endeavour within each PIC as well as for the region. This can be achieved through public, private, and academic sector initiatives to draw in all sectors of government as well as the relevant UN agencies and regional PIC-representative organisations.

## 1. Introduction

Child-specific environmental health indicators are required to track progress on children’s health because children are a vulnerable, valuable, and at-risk subset of the population [1]. The theme of this review is the application of indicators for children’s environmental health in a particular setting, namely Pacific Island Countries (PICs).

Children are much more vulnerable and at risk to environmental hazards than adults because they breathe, drink, and eat much more than adults relative to body weight, resulting in greater exposure to environmental hazards present in air, water, and food [2,3,4] that can occur in the different environments in which children find themselves every day [5].

The World Health Organization (WHO) has developed children’s environmental health indicators (CEHI) for physical injuries, insect-borne disease, diarrheal diseases, perinatal diseases, and respiratory diseases [6] and provided implementation strategies with global partners to facilitate country-level uptake of these indicators across the WHO regions [7]. However, the uptake and implementation has been incomplete [6].

Given the considerable economic, environmental, and social challenges the PICs and their children face, questions can be asked about the extent to which CEHI have been developed and implemented across the PICs.

## 2. Children’s Environmental Health in Pacific Island Countries

Children of Pacific Island Countries (PICs) are of the most vulnerable in the world. As small island developing states (SIDS), PICs share the vulnerabilities of other SIDS but are also potentially more vulnerable and are therefore at increased environmental health risks because their own constituents are often very remote (populations of the same country spread over many distant islands), with little direct links to nearby larger economy states, and they often rely on foreign aid for economic support [8].

Children have a particularly special status in the PICs [9], recognized perhaps some time before the wider global realization of the vulnerabilities and risks as well as the value of children, as reflected by the Global Initiative on Children’s Environmental Health Indicators, which was launched at the World Summit on Sustainable development in September 2002 [1,7]. The 1995 Yanuca Declaration implies that healthy islands support the health and wellbeing of children and the environment and thus play a cardinal role in the future of PICs [9]. At the Eleventh Pacific Health Ministers Meeting in Fiji, held in April 2015, the meeting reaffirmed the Healthy Islands vision. The Healthy Islands concept, as articulated by the Yanuca Declaration, has five elements—all of which are of importance in the context of children’s environmental health (CEH):

- Children are nurtured in body and mind;
- Environments invite learning and leisure;
- People work and age with dignity;
- Ecological balance is a source of pride; and
- The ocean is protected.

Holistically then, the Healthy Islands vision embraces the all-important role that environment plays in supporting and protecting the health of people—especially the children—of the region.

The PICs naturally place strong emphasis on the role that sustainable environments play in their health and wellbeing, prosperity, and protection [9]. However, small island developing states are highly environmentally vulnerable, especially because of the effects of climate change, low economic growth [10], and the local environmental impacts of activities such as forestry and mining [11]. The PICs, with their high birth rate and high proportion of young people [12], are very aware of the need to protect and nurture their children.

CEH as a discipline has emerged over the last few decades because of our need to better understand and manage an increasingly complex set of issues regarding child health, but, as such, it is not yet widely considered, nor practised, in PICs.

CEH is based on a wide and encompassing context of ‘environment’, as it considers and informs environmental and public health practitioners and decision-makers about how early life exposures to environmental and social conditions (which can be detrimental and/or beneficial) influence the health and development of children either positively or negatively. CEH practice applies a very inclusive definitions of childhood (broader than the Sustainable Development Goal (SDG) definition of childhood which includes ages 0–14 inclusive), in order to cover exposure of children to environmental conditions during the ‘windows of vulnerability’ that occur during pregnancy, infancy, childhood, and adolescence [5]. As such, CEH also includes maternal exposures to environmental conditions prior to conception that may influence the health of children. These windows of vulnerability have no counterpart in adult life. Exposures to harmful environmental influences during these sensitive periods program the child’s body toward diseases that may not become diagnosed until much later in life [2,4,5].

Whilst the broader practice and services of environmental health are aimed at managing all aspects of environment that can be to the detriment/benefit of whole populations across all sectors of society and government, a more specialist practice of CEH needs attention in order to manage children’s unique vulnerabilities, risks, and value.

The environments in which children live, learn, play, and even work have a particular significance, as children need protection during the times they spend in them. These environments include the inter-uterine environment (for the duration of pregnancy), home (depending on age—ranging from 70–100% of time), travel, care-giving, school, occupational, and neighbourhood—the latter strongly associated with the built (design and layout of urban areas) and natural (green space) environment [2,3,5,13,14,15].

Children are disproportionately exposed to adverse environmental conditions compared to adults. In relation to body weight, children drink more water, eat more food, and breathe more air than adults. Children’s metabolic pathways, especially in the first months after birth, are immature. Thus, if these environmental components are not of acceptable quality, their abilities to fend off, metabolize, detoxify, and excrete many environmental pathogens and toxins are less than those of adults. Additional age-dependent characteristics such as their hand-to-mouth behaviour and the fact that they live and play close to the ground further increase their environmental exposures. Children are less able than adults to deal with adverse environmental hazards (e.g., disaster, changing climate) and often cannot avoid exposures—making them more susceptible to injury [2,3,4,5,13,15,16].

With limited resources and many competing priorities, PICs may find it difficult to identify and improve the key attributes of the environments in which their children and young people could survive, thrive, and transform as envisaged by WHO and UNICEF [17,18]. Environmental health indicators (EHIs) specifically focused on children over the stages of their development to adulthood are a tool that can be used to enable PICs to improve their children’s environmental health through measurement, monitoring, and targeted action [19].

CEHI for PICs must inform on the common but also on the unique environmental influences (including risks) on children’s health in early life, whilst also considering social and economic pressures. This implies the need for indicators directly associated with maternal exposures and the various anthropogenic environments with their economic and social conditions in which children live, learn, play, and work, all of which affect children’s health. This calls for very specific—perhaps unique—indicators to guide decision-making about the health and environment of children in the PICs. These indicators will lead us to understand the current and future status and risks and benefits of environmental hazard and protection as well as the exposures of mother, infant, toddler, child, and adolescent health in a fast-changing environment—thus reflecting environmental health effects through all stages of childhood. This will enable us to plausibly forecast the future environmental, social, and economic burden of disease [20].

CEHI for PICs must inform about a more complex and broader environmental context than most other countries would have to consider—even more so than the traditional environmental health risk factors often described for environmental and public heath practice [19]. This is because children’s environments in PICs are substantially layered and complex. Their environments are naturally changing over space and time; current-day environmental changes for PICs are rapid and unprecedented because of (amongst others) demographic shifts to urbanisation, climate change, rising oceans, local and imported pollution, development pressures, ageing infrastructure, and more [10,11,14,21,22]. Among these shifting environments, CEHI for PICs must also inform about multiple exposures at different life stages, which are also rapidly transforming because of demographic shifts in communities and developing economies [10]. Lastly, CEHI must inform about the influences of the environment on the human genome and epigenome [5], as these will have substantial impacts on PICs via the longer-term burden of environmentally-related diseases.

CEHI that measure children’s unique windows of vulnerability should be developed and actioned. A challenge of CEHI is that health effects of exposures during childhood may not be seen for many years. Collection of data on these exposures is an investment for the future, enabling research into linkages between maternal health and child health, complex cause-effect chains, and health outcomes with long lead times.

This review—given the complexities highlighted above—explores the extent of indicator development and operational uptake to inform about children’s wellbeing in the context of their environments in Pacific Island Countries. While it does not propose new indicators, it reflects (with appropriate examples) on the areas and potential for developing new and more targeted indicators to address issues related to environment and child health in Pacific Island Countries. 

## 3. Methodological Approach

We conducted a systematic search of relevant documented information using the general Internet, as well as the databases of PubMed and Google Scholar and relevant UN agency databases, to find publicly available Pacific Island Country information. We reviewed the available literature of the three UN agencies that align closely with the concept of CEH—namely WHO, UNICEF, and UNEP. We also investigated regional literature (compiled with or without UN agency support), for instance, the Secretariat of the Pacific Community (SPC).

We first searched for existing CEHI, EHIs, initiatives, and reports specific to PICs and then extended our search to global CEHI information.

We also reviewed the specific indicator frameworks that could reflect environmental causes, exposures, and health effects that were suitable for use in PICs. Whilst there are several descriptions of what CEHI could look like and what would be likely frameworks to present these [6,19,23,24], we re-contextualised what these would mean for Pacific Island Countries that place special emphases on child health and the environment [25].

We then selected contexts and environmental causes appropriate to expressing the relationship between children (over the childhood to adolescent lifespan) and their environmental as well as social conditions, their exposures and behaviours, and the likely health effects. We also explored the practicality of indicator clusters, that should be considered for the development of an indicators suite.

## 4. General Findings

While most PICs deliver on a variety of environmental health services—aspects such as housing/shelter, water, food, sanitation, and waste management services—we were unable to find any monitoring or information systems that measure and report on the efficiency of delivering these services. The lack of systems and/or the need to establish information sharing systems on content, impact, and future direction of health and environment are noted in a substantial number of the reviewed literature [2,14,26,27].

It follows that indicator frameworks for environmental health services could not be found. More specifically, we were unable to find any CEHI specifically developed for one or more PICs. Whilst there are a number of reports and initiatives that highlight the need for EHI and CEHI, the detailed work of determining a set of CEHI for adoption and setting up a monitoring and measurement framework has not been completed in PICs. This is understandable, as information systems are essentially based on indicators and their measures.

We found a limited number of peer-reviewed reports of studies on environmental health indicators through academic database searches. This may be because the development of environmental health indicators is often led by governments or international organisations, which results in most documents being published as grey literature. This lack of peer-reviewed literature has been noted by other studies [23].

The PICs are not unique in this instance. A 2014 study [28] found that EHI initiatives were not yet well established globally, especially in developing countries. There were very few operational Environmental Health Indicator programmes either at national or regional scales and those that did exist were mainly found to be for developed countries. The indicators included in the programmes were limited both in terms of their position in the causal chain and in terms of their thematic scope. The use of indicators was also limited by uncertainties in framing the exposure-response relationships that they implied, and the consequent inability to translate the indicators into common measures of impact, be it on the environment, exposure, or health side. In addition, there was no information on the extent to which the indicators have been applied in decision-making. Most were exposure-side indicators focused on sanitation, water, and air quality [28].

Within the PICs, the Healthy Islands monitoring framework [27] contains some health-side indicators that are specific to children and also some optional ecological balance indicators related to the framework.

Looking outside the PICs, we found just two recent studies that specifically proposed indicators for CEH [6,23]. In terms of governance systems, we found one active operational children’s environmental health monitoring system. This is produced by the Children’s Environmental Health Network and contains data specific to the USA. It uses hazard, exposure, health effect, and intervention indicators [29]. There are a number of regional and national environmental health monitoring systems including the European Environment and Health Information System (ENHIS), the USA National Environmental Public Health Tracking Network (NEPHTA), and Environmental Health Indicators New Zealand (EHINZ) [30,31,32]. These systems contain some information specific to children, but they are all for developed countries.

One further source of potential CEHI is the UN’s Global Sustainable Development Goals (SDGs). These consist of 17 interrelated goals, supported by targets and indicators. Many are relevant to children’s environmental health, but to make them CEHI-specific, country-level data collection for SDG progress tracking would need to be disaggregated into subsets such as age, gender, and geographic location. If collected, this information could contribute to country-level reporting to the UN [33], as well as catalyse in-country CEHI development.

## 5. Childhood Vulnerabilities, Risks, and Values in the Context of Their Environments

It was clear from the review that indeed all population age groups are, to variable extents, subject to exposures to environmental conditions. Where these conditions are hazardous, the health outcomes will invariably be negative. This is the case globally and not just for PICs. The literature also shows, however, that children require specific considerations for monitoring their environmental exposures. This will require a specific set of indicators for children’s environmental health. The rationale is to address specific child vulnerabilities, risks, and values across childhood development stages within the context of the important environments, behaviours, hazards, exposures, and health outcomes, rather than just disaggregating general environmental health data into age clusters [1,19].

Children’s particular vulnerabilities also increase their risk of adverse health outcomes. Depending on the life stage of a child (i.e., in utero and during the first years of life), they do not have the capacity to avoid environmental risks [1,5,19]. In the context of environmental health indicators, children also have specific values. The longer-lasting public health investments in their health over their life spans are substantial—a sick child will cost society more if they develop lasting morbidity into adulthood [4]. From a social-economics perspective, child health outcomes associated with environmental risk will be useful to model and forecast future economic burden of disease and facilitate preparedness of the governance systems that have to deal with these outcomes. Children’s environmental vulnerabilities also make them sensitive early warning indicators of environmental health threats to populations [1,19]. Vulnerability and risk as well as value are key incentives for developing and implementing child-specific environmental health indicators at the country level.

Their stages of development place children in specific environments that environmental health practices in PICs do not adequately consider nor attend to. For instance, environmental health law and governance in most PICs tend to focus on delivering on the issues that should mitigate on the environmental hazard side—such as water, food, sanitation, environmental hygiene, and waste management—with the understanding that this will have a positive influence on the health outcome side. Such services often do not specifically consider the efficiency of their reach to influence and protect child health.

To provide this specific context, Table 1 summarises childhood development stages, the important environments and the health risk posed by each environment, environmental behaviours, hazards and exposures, as well as health outcomes. While there is information on child activities and associated risks in home and other care environments, important factors such as choice, time spent in these environments, governance, and responsibility for each of the child environments are not addressed in literature, which is a serious gap in the knowledge required to develop suitable indicators. We nevertheless—in Table 1—provide a very broad risk classification for the child environments based on perceived risk posed by each environment, as well as the estimated time that children might spend in each environment.

## 6. Requirements for Children’s Environmental Health Indicators for PICs

Frameworks for understanding and practising environmental health have been under constant review and application for many decades [6,19,23,24]—especially since the Earth Summit in Rio De Janeiro in 1992. These frameworks—with some variations—mostly apply a cause, exposure, and health effect model [36]. More recently, the concept of planetary health has been framed with a similar approach, using causal drivers that exacerbate mostly socio-economic factors, leading to adverse health effects [37].

We developed a simple but novel framework along these lines, consisting of distal (underlying) drivers, the changes these drivers affect in the environments of children, the conditions and commodities in these environments that will be affected, and how these conditions and commodities, as well as behaviours, shape children’s exposures that will ultimately lead to adverse health outcomes. This will provide a clear framework for community-level indicator development and will enhance the incorporation of these indicators into environmental health information services. Table 2 presents a narrative summary of the framework organized as five indicator clusters.

## 7. Sectoral Origin of the Information

The data that will define the CEHI will have to come from many sectors in the PIC society [11,39]. CEH is highly inter-disciplinary and cuts across paediatrics, epidemiology, occupational and environmental toxicology and medicine [23,27], industrial hygiene, exposure science, engineering, architecture, urban planning, social work, education, ecology, economics, and political science [5,11,19].

A comprehensive level of data will be needed to inform indicators—a requirement that is already difficult for developed countries [6,39] and almost impossible for developing regions such as small island developing states [10,16,18,30]. However, some data is already being collected though monitoring and surveillance in the PICs by the various sectors. Global agencies such as the WHO, UNDP, UNEP, and UNICEF support gathering of data by ministries responsible for health, environment, economy, development and planning, local government, transport, tourism, mining, energy, and more. Aid organisations, universities, industry, and commerce all collect data to some extent. Global and regional data (e.g., satellite data), be it for collected for other purposes or available from international organisations such as the Secretariat of the Pacific Community (SPC) and Secretariat of the Pacific Regional Environment Programme (SPREP), can be used. A suitable set of CEHI, be it for local or regional purposes, will drive the necessary collaboration to integrate much of this data—useful as is, or redirected and repurposed,—and provide incentives for increased targeted monitoring and surveillance.

## 8. Discussion

The key to a peaceful, prosperous, and sustainable world is healthy, safe, educated, and empowered children and young people [40]. Children are especially vulnerable and at high risk of environmental causes of adverse health effects. Children’s unique sensitivity to environmental changes are in themselves valuable indicators, as it helps us better understand the current health risks. At the same time, understanding what is happening to children in the context of environmental causes of disease is not just about understanding the current burden of disease, but also the future burdens, since many of the effects on children’s health are carried forward into adult life, thus adding additional disease burden [1,4].

While these are sound arguments, it is noticeable that the implementation of CEHI by countries globally has been slow, given that many countries have the means to make it possible for their children to thrive. Many countries, including developing countries, however, often do not fully recognise the support role that the environment can play in supporting sustainable development for the health of women, children, and adolescents [18]; they might also not have the means. The PICs, with few institutional, governmental, or philanthropic resources to help them, are some of the most vulnerable countries in the world to rapidly changing environmental conditions, created by globally developed world carbon emissions and by population growth, leading to rapid urbanisation and growth of informal settlements and pressure on natural resources [37].

As countries develop economically, children’s environmental health can be expected to improve [41]. However, without a strong environmental health information service, based on a well-developed set of CEHI, a consequence of development can be greater inequalities in children’s health as living conditions and health service coverage decrease in the poorest populations in developing countries [42,43]. Therefore, it is of critical importance to measure both lead and lag (environmental cause and health outcome) indicators in order to capture the past, current, and predicted future of children’s environmental health in PICs. This will enable PICs to avoid unintended health as well as other social and environmental consequences of economic development or overseas development aid investments.

The PICs, in common with other countries, collect information that can form part of a set of CEHI. The reality is that any relevant measurements have been incidental to other initiatives across many different domains and sectors of PIC society and have not been integrated into proper information systems. While children’s environmental health is an emerging discipline that aligns with, and can also be a subset of, environmental health services in PICs, the development and use of a CEHI suite will provide a strong impetus for intersectoral collaboration and will support decision-makers to understand environmentally-related future burdens of disease.

Whilst we argue that a well-developed CEHI suite will track progress from the community level up through to the higher-end of decision-making at government level and thus enhance the targeting of economic, social, and environmental priorities, the development of a comprehensive set of indicators will involve a complex process. Most importantly, the development, implementation, and collection of indicators must be achievable and tailored to each country’s specific needs. To improve the likelihood of inclusivity and success, methods such as systems thinking, participatory modelling, and elicitation of expert opinion will have to be applied. Perceptions and opinions from all stakeholders about environmental health problems in PICs must be captured. From this input, the priorities to be addressed and the actions required to solve the problems can be determined. Following on, development of the various levels of indicators will then be undertaken, enabling measurements of progress and impact to inform decision-makers.

We do not prescribe nor suggest what the actual indicators should look like that emerge from this complex process. Instead we propose the broad requirements to be considered for developing indicators and their measures. CEHIs must be appropriate—for instance, indicators that measure poverty, informal settlement populations, internal and external air quality in rural, urban, and peri-urban contexts, ocean and forest health, and access to environmental health services are universally important but must be tailored to PIC-specific circumstances and needs.

In terms of child vulnerabilities and risks in the context of CEHI, the sections in our framework where indicators for these will be developed are the drivers, changes, conditions, and exposures. These are the areas where the more active interventions towards mitigations are foreseen. The disease and injury section will provide the value indicators, which will be used to track progress of interventions towards, as well as forecast the future of, health and wellbeing outcomes for children.

Sustainability is the key to maintaining healthy, ecologically balanced environments to support the health and wellbeing of PIC children. This is clearly recognised in the ‘Healthy Islands’ framework [27]. Adverse human impacts on natural systems have accelerated steeply since the 1950s and current rates are deemed unsustainable. On the premise that the need for sustainability is self-evident to protect the wellbeing of children, sustainability measures should be included across all information gathering by the various sectors. Because of the sensitivities it addresses, CEHI for PICs will raise awareness and ensure that the data collected through the use of CEHI can be modelled to forecast future environmental health risks and impacts as well as support country-level reporting on the SDGs.

Finally, development and implementation of CEHI must in the first instance be driven at the communities and local government level. A probable reason why there has been slow progress of development and implementation for CEHI in PICs is failure to translate and integrate the many excellent policy-level environmental health indicators proposed by support agencies to action at the community services level. This review has again enabled us to revisit the requirements for children’s environmental health indicators that are practical, multi-sectoral, and address the vulnerabilities, risks, and value of children in the Pacific Island Countries.

## 9. Conclusions

This review has shown that whilst child-specific environmental health indicators are essential to track progress of children’s health and wellbeing over their life span, these do not exist nor is it practised in Pacific Island Countries. Children’s environmental health indicators will be essential for supporting Pacific Island Countries to achieve their Healthy Islands vision of environments that nurture children in body and mind. The two tables were designed to focus on the unique circumstances of vulnerability, risks, and value of children for environmental health practices in Pacific Island Countries. They further highlight why we must develop child-specific environmental health indicators.

We recommend that the setup and measurement of CEHI be implemented as a fundamental integrated multisectoral activity for Pacific Island Countries. It will provide a tool for prioritising global and local issues that influence children’s environmental health, as well as for focusing local and global attention on their most pressing problems. The framework of indicator requirements and examples of indicators we propose highlights the priorities and areas of multisectoral collaboration in Pacific Island Countries to protect and improve children’s environmental health.

## Figures and Tables

**Table 1:** Childhood environmental health risks by development stage.

Development Stage	Child Health Risk Posed by Child Environment ^a,b,c^						Environmental Behaviours	Environmental Hazards and Exposures	Health Outcomes
	H	T	CCAH	S	O	N			
**Pre-birth**	XXX	XXX	X	X	-	XX	Maternal and paternal exposures	Poor nutrition, smoking, hazardous chemical intakes, environmental stress	Foetal exposures, birth weight anomalies, congenital abnormalities
**Infancy** Birth to 12 months	XXX	XXX	X	-	-	XXX	Move in ‘floor’ zone, hand to mouth behaviour	Poor indoor and outdoor air quality, poor water and food quality and quantity, cleaning products and pesticides, poor sanitation and environmental hygiene, stressed environments	Diarrhoea, parasitic diseases, environmental enteropathy, malnutrition, stunting, respiratory diseases, neuro-developmental abnormalities, poisoning
**Toddler** 1–3 years	XXX	XX	X	-	-	X	Above, plus increased mobility and exploration, no apprehension of danger		
**Pre-school Child** 3–5 years	XXX	XX	X	-	-	XX	Above, plus increased exposure to other children, people, and wider environment		Respiratory diseases, malnutrition, weight abnormalities, parasitic and vector-borne diseases, reduced learning capabilities.
**School-aged Child** 5–12 years	XX	-	-	XX	-	X	Increased learning, group activities, peer pressure		
**Adolescent** 12–19 years	XX	-	-	XX	X	XX	Increased sense of adventure, peer pressure, increased risk taking	All of the above plus occupational hazards and exposures, increased UV exposure	Social and behavioural problems, overweight, injuries

^a^ Legends. H: Home; T: Walk/travel away from home; CCAH: Child care away from home; S: School; O: Occupational; N: Neighbourhood. ^b^ Health risk considerations. 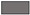 High risk of impact; 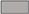 Risk of impact; 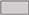 Lower risk; 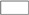 No monitorable risk. ^c^ Time spent in environment. XXX: Up to 100% of time spent; XX: Up to 60% of time spent; X: Up to 30% of time spent; -: No time considerations. Compiled from [4,5,16,19,22,34,35].

**Table 2:** Children’s environmental health indicator clusters, their indicators’ requirements, descriptions of indicator types, and broad examples of indicator measures.

Indicator Clusters and Requirements	Indicator Type	Examples of Measures
**Underlying (distal) drivers—to more plausibly predict the local conditions**		
While CEHI packages will be developed for mostly local conditions, indicators of global and regional environmental changes, economic development and social patterns are useful predictors of similar local patterns. For instance, new developments in industry, agriculture and forestry are often emulated locally but might not be suitably adapted for local conditions. Distal environmental change indicators, usually dominated by changing climate and global pollution, will provide predictors for local environmental change.	Economic, demographic, environmental change.	Global GDP, foreign aid, demographic distribution trends, global climate change, regional environmental measures.
**Environmental change—to determine the trends that will increase vulnerability and risk**		
Changes that the underlying drivers bring on in the local environment come from mainly the things that we live by and the wastes and depletions that result—and all will affect child wellbeing. For PIC, this also includes the real threat of natural disasters, which are occurring more frequently. Global climate change is driving local weather patterns, more frequent disaster events and changes in the oceans (rising sea levels) and reefs of the Pacific. Local patterns of consumption and need/ease of travel (transport) force energy production, resource depletion (oceans and forests) and proliferation of imported goods. This also lead to local waste generation and pollution of air, freshwater, ocean, food and forests as well as increased proliferation and new habitats for insect vectors. New, seemingly more efficient goods such as vehicles and fuels, are imported at increasing rates often without consideration for the unintended impact they might have. A classic example is motorised vehicles—these are all imported and invariably become local waste at some point, with little if any means to remove or having available land to dispose of the wastes properly. Derelict vehicles become hills of hollow waste—providing new habitats for insect disease vectors. Social drivers such as demographic shifts (population growth, younger populations, urbanisation), cultures and behaviours and education all contribute to local environmental change. While these are not unique to PIC, these are more challenging (and to a large extent distal) in PIC because of vast distances between islands of the same country, low levels of resources to mitigate against environmental change, and modes of travel between the islands to make things work (governance). Rapid urbanisation leads to challenging settlement conditions and neighbourhoods—loss of habitat often with a lack of green space - that do not protect child health. Biodiversity loss is driven by economic development and land use changes for agriculture and urban development. All of these changes have a direct influence over the environmental conditions and environmental commodities that children are subject to, summarised in the next section. If the changes are not adequately managed towards health protection, then the conditions and commodities will lead to adverse exposures.	Natural Environmental condition, education/culture/maternal education, energy usage, insect vector counts and habitats, condition of the built environment, urbanisation, air, water soil and food pollution, waste management, the use of chemicals such as fertilisers and pesticides.	Disaster frequency/magnitude, local sea level rise and loss of habitat, local ocean acidity, local ambient and ocean temperatures, reef health, biodiversity loss measures, fish stocks, marine reserves, deforestation trends, land use changes for urbanisation, industrial development (tourism and mining) and agriculture. Proxy for pollution measures—these can be fossil fuel usage—both for locally generated and imported energy, vehicle numbers disaggregated by type and fuel. Measures of proportion of land inhabited by insect vectors, urbanisation and settlement growth, contaminated land from industrial activity.
**Environmental conditions and commodities that will indicate the environmental hazard areas**		
These are essentially the ‘Healthy Environments’ required to protect the health and wellbeing of children. These conditions are measurable and include ventilated and adequately-serviced and safely constructed buildings (with sanitation and waste disposal) for home, care away from home, school and workplace, in hygienic environmental surroundings (in buildings and neighbourhoods) preferably with ample green space. This will require accessible and available safe water and food. Soils must be kept free of anthropogenically induced microbial pathogens, parasites and chemicals. Such protected and serviced environments will also prevent vector proliferation and movements. Society must transition to clean energy, reduce vehicle transport and properly maintain roads—this will reduce air pollutants such as particles and gases. The proper use of vehicles and electric good will reduce noise, heat, radiation as well as reduce risks of injuries. Properly maintained environments will also reduce risks of residual mental stress on children—especially with the constant and unavoidable risks of sudden changes in environment due to disaster, or longer term but clearly apparent stressors such urbanisation, loss of habitat and rising oceans.	Housing and building quality. Quality of domestic and outdoor environments including green space, availability and quality of water food and sanitation. Hazard -reducing services such as waste management including imports and downstream waste reduction. Use of household chemicals and pesticides, Vector distribution	Air quality measurements; children living/learning/being cared for in environments with clean fuel stoves/improved biomass fuel stoves, and/or where at least one adult smokes, measures of water quality and availability for children’s environments—drinking and play/bathing, no access to basic sanitation/environmental hygiene services, access to green space, indices of households providing conditions for insect borne disease transmission.
**Child-specific behaviour factors to determine exposure and risk and predict impact**		
The extent to which adverse environmental conditions and commodities pose detriment to children depend on the actual conditions, but is also driven by their unique behaviours. Properly managed exposure-related behaviours of children and their carers will enhance the benefits of properly maintained environments and reduce the risks and impacts of associated diseases. Age-related behaviours of children that influence their exposures are summarised in Table 1 above. Requirements for sound and healthy environmental conditions and commodities will be determined primarily by the child’s stage of development. For instance, crawlers and toddlers require clean lower-zone domestic environments because they crawl, sit, stumble and mouth things with which they come in contact. Children living in informal settlements have multiple exposures. In addition to general air and water pollution, they may also spend time playing or scavenging in areas polluted by solid and industrial wastes. Behaviour change might not be sufficient—as the economic condition of the household will determine the exposure. Children living in close proximity to busy roads are exposed not only to air and noise pollution but also to risk of death or traffic injury. Children living in slum conditions have little choice but to interact with that particular set of environments. As children become more mobile, they explore spaces accessible from the home. Green spaces therefore are important for physical and cognitive development but might not be available for substantial numbers of children. Whilst the level of nutritional exposure will in part be determined by the quality of product they receive from their environment—it is also childhood eating patterns that largely determine how optimal their nutritional exposure will be. This behaviour, in combination with appropriate personal hygiene, depends much on parental knowledge and practices in raising the child at home and elsewhere. At school, the education systems must ensure that sound nutritional as well as environmental knowledge and practices are embedded in the child. Children often have to work out of necessity—with the workplace often not adapted to the vulnerabilities and risk of a young person.	Parental/carer/child education, child education, behaviours, child activities. Proximity to inevitable hazardous conditions i.e., underdeveloped neighbourhoods and proximity to roads.	Behaviour-related exposures to wastes in neighbourhood or through scavenging, proximity to a busy road, parental/carer education to appropriate school level; school age children attending school, children completing primary/secondary education, children in the workforce
**Disease conditions and injuries to understand current impact and forecast future social, economic and environmental burden of disability**		
The major diseases that children risk contracting through adverse environmental conditions and exposure behaviours are summarised in Table 1. In terms of data requirements, the PIC Health sectors should have adequate primary data which can be translated to appropriate indicators and their measures. In turn, a well thought through implementable Children’s Environmental Health Indicator sets will point out gaps in the health data as well as provide the data that will be used to model the future burdens of disease.	Maternal health/perinatal mortality, child mortality and morbidity for chronic and communicable disease, child injury including poisonings	Mortality rates with causes disaggregated into acute respiratory illness, diarrhoea, insect-borne disease, NTDs, physical injuries, other. Chronic disease prevalence including respiratory disease and communicable disease prevalence including acute respiratory illness, diarrhoea, NTDs respiratory disease. Incidence of physical injuries and poisonings reported disaggregated by age band, urban/rural/informal settlement

Compiled from [1,2,6,14,19,25,27,34,35,38].
